# Evaluation of *Ilex guayusa* and *Piper marginatum* Extract Cytotoxicity on Human Dental Pulp Mesenchymal Stem Cells

**DOI:** 10.3390/dj12060189

**Published:** 2024-06-20

**Authors:** Luis G. Sequeda-Castañeda, Luisa F. Suárez-Carvajal, Mayra A. Téllez-Corral, Sandra J. Gutiérrez-Prieto, Henry A. Méndez-Pinzón

**Affiliations:** 1 Department of Chemistry, School of Sciences, Pontificia Universidad Javeriana, Bogotá 110231, Colombia; lsequeda@javeriana.edu.co; 2 Oral Rehabilitation, School of Dentistry, Pontificia Universidad Javeriana, Bogotá 110231, Colombia; luisa_suarez@javeriana.edu.co; 3 Dentistry Research Center, Pontificia Universidad Javeriana, Bogotá 110231, Colombia; tellezm@javeriana.edu.co; 4 Department of Physics, School of Sciences, Pontificia Universidad Javeriana, Bogotá 110231, Colombia

**Keywords:** dental remineralization, fluorine, *Ilex guayusa*, *Piper marginatum*, human dental pulp stem cells (hDPSC), cytotoxicity, Raman spectroscopy, nanobiochips, SERS effect, fluorescence microscopy, fluorescent activated cell sorter (flow cytometry)

## Abstract

Background: Amelogenesis imperfecta is a hereditary disorder affecting dental enamel. Among its phenotypes, hypocalcified AI is characterized by mineral deficiency, leading to tissue wear and, consequently, dental sensitivity. Excessive fluoride intake (through drinking water, fluoride supplements, toothpaste, or by ingesting products such as pesticides or insecticides) can lead to a condition known as dental fluorosis, which manifests as stains and teeth discoloration affecting their structure. Our recent studies have shown that extracts from Colombian native plants, *Ilex guayusa* and *Piper marginatum*, deposit mineral ions such as phosphate and orthophosphate into the dental enamel structure; however, it is unknown whether these extracts produce toxic effects on the dental pulp. Objective: To assess cytotoxicity effects on human dental pulp stem cells (hDPSCs) exposed to extracts isolated from *I. guayusa* and *P*. *marginatum* and, hence, their safety for clinical use. Methods: Raman spectroscopy, fluorescence microscopy, and flow cytometry techniques were employed. For Raman spectroscopy, hDPSCs were seeded onto nanobiochips designed to provide surface-enhanced Raman spectroscopy (SERS effect), which enhances their Raman signal by several orders of magnitude. After eight days in culture, *I. guayusa* and *P. marginatum* extracts at different concentrations (10, 50, and 100 ppm) were added. Raman measurements were performed at 0, 12, and 24 h following extract application. Fluorescence microscopy was conducted using an OLIMPUS fv1000 microscope, a live–dead assay was performed using a kit employing a BD FACS Canto TM II flow cytometer, and data analysis was determined using a FlowJo program. Results: The Raman spectroscopy results showed spectra consistent with viable cells. These findings were corroborated using fluorescence microscopy and flow cytometry techniques, confirming high cellular viability. Conclusions: The analyzed extracts exhibited low cytotoxicity, suggesting that they could be safely applied on enamel for remineralization purposes. The use of nanobiochips for SERS effect improved the cell viability assessment.

## 1. Introduction

Amelogenesis imperfecta is the term assigned to a group of clinically and genetically heterogeneous conditions primarily affecting enamel structure, along with other oral and extraoral dental tissues [1,2,3]. It results from alterations in mineral apposition, such as calcium, phosphorus, and fluoride during the amelogenesis transition period. Amelogenesis imperfecta is classified into four types, primarily based on the phenotype and mode of inheritance. The hypoplastic type occurs during the secretory phase of amelogenesis, resulting in thin enamel and surface defects, such as grooves, furrows, and pits. The hypomineralized type is characterized by normal amounts of enamel matrix, although poorly mineralized. The hypomaturative type presents a mottled appearance on dental enamel, generally white in color, altering its translucency in localized areas. Last, the hypoplastic–hypomaturative type, which presents a combination of these two phenotypes, is associated with pulp chamber elongation and short roots, characteristic of taurodontism [4,5].

Dental enamel defects and their remineralization process have evidenced the existence of substances with the ability to integrate minerals, such as fluoride, calcium, and phosphate into the enamel structure [6,7,8]. In preventive and conservative treatments, fluoride is used as a reference mineral, as it strengthens tooth enamel and helps prevent dental caries [9]. However, excessive fluoride consumption can cause health problems [10,11,12,13,14,15,16,17,18,19,20,21,22,23,24,25,26,27,28,29]. Fluoride toxicity usually occurs when extreme amounts of fluoride are ingested through drinking water [19,21], fluoride supplements [30,31], toothpaste [32], or in extreme cases, by ingesting products such as pesticides or insecticides that contain fluoride [33]. Excessive fluoride intake can lead to a condition known as dental fluorosis [34], which manifests as stains and discoloration on the teeth [20]. In severe cases, fluorosis can damage teeth and affect their structure [12], causing skeletal alterations [20,35] such as an increase in osteoporosis and bone fracture prevalence [36]. Furthermore, high fluoride exposure has also been described to reduce IQ levels in children [36,37,38,39].

In addition to dental fluorosis, chronic exposure to high levels of fluoride can cause other adverse health effects [15,16,17,18,23,31], such as generating bone problems [15], causing liver and kidney damage [16], affecting the gastrointestinal system [40], and inducing neurological disorders [10,11,12,13,14,15,16,17,18,19,20,21,22,23,24,25,26,27,28,29,31,32,34,35].

Medicinal plants provide natural alternatives for dental care. For example, tea tree, sage, chamomile, and neem exhibit antibacterial and anti-inflammatory properties that can promote oral health and prevent certain dental diseases [41,42,43,44,45]. Several studies have reported *Galla chinensis*, a Chinese plant, contains tannins and flavonoids that allow for enamel matrix remineralization [46,47]. In Colombia, some plant species containing secondary metabolites such as flavonoids and tannins, similar to those reported for *G. chinensis*, have been identified [48]. *I. guayusa* [49,50] and *P. marginatum* are among these plants [51,52], for which several traditional medicinal uses have been reported [50,53,54,55]. A previous study by our group highlights the use of these extracts as antimicrobial agents in periodontal disease [56]. A recently published study demonstrated that extracts applied to sections of hypocalcified amelogenesis imperfecta teeth deposited minerals such as phosphate and orthophosphate, which are key components of hydroxyapatite [57].

It is noteworthy that although medicinal plants are considered safe and effective, they can be toxic, especially during pregnancy and in children, and their use without proper supervision can lead to serious poisoning. Some plants at high doses may have undesirable side effects or even be toxic [58,59,60]. Additionally, some individuals may be allergic to certain plants [61,62,63]. For example, *Jatropha curcas* generates abdominal pain, diarrhea, and vomiting [64]. *Callilepis laureola* causes abdominal pain, semi-coma, anxiety, vomiting, diarrhea, fatal hepatic necrosis, and death [64]. *Datura stramonium* is associated with symptoms of mydriasis, tachycardia, agitation, disorientation, delirium, hallucinations, and restlessness [64]. *Gelsemium elegans* generates muscle spasms and convulsions and causes death due to depression of the nervous and respiratory systems [65]. *Cassia occidentalis* causes acute liver failure, muscle damage, and encephalopathy [65]. Aristolochic acid, the major compound in *Aristolochia* species, is nephrotoxic and carcinogenic [65].

Therefore, it is important to conduct cytotoxicity, cell viability, and acute and subchronic toxicity studies, among others, using laboratory cell lines and animals. To this end, various analytical techniques and methods are available, including spectroscopic, colorimetric, fluorometric, and bioluminescent approaches. These include Raman spectroscopy, fluorescence microscopy, flow cytometry, MTT assay, trypan blue exclusion assay, resazurin assay, LDH assay, DNA dye cytotoxicity assay, real-time cell viability assays, ATP cell viability assays, live-cell protease viability assay, tetrazolium reduction cell viability assays, resazurin reduction cell viability assay, dead-cell protease release cytotoxicity assay, lactate dehydrogenase (LDH) release cytotoxicity assays, and DNA dye cytotoxicity assay [61,66,67,68,69,70,71].

Raman spectroscopy serves both to assess cell viability and to analyze the chemical composition and molecular structure of biological samples. This non-destructive, non-invasive technique generates a unique chemical fingerprint of living and dead cells. Surface-enhanced Raman spectroscopy (SERS) significantly amplifies the Raman signal, enabling compound detection at low concentrations using a nanostructured metal substrate [70,72,73,74,75,76,77,78,79]. In contrast, fluorescence microscopy is employed in the study of biological processes at the molecular and cellular levels, offering subcellular resolution and the ability to observe living systems. It utilizes advanced fluorescent dyes to differentiate between living and dead cells based on various cellular properties [80,81,82]. Flow cytometry analyzes the physical and chemical characteristics of individual cells using light scattering and fluorescence as they pass through a laser. It provides detailed information on cell size and morphology, and with the use of fluorescent markers, it is possible to perform comprehensive studies on viability, protein expression, and more. This technique is efficient for rapidly analyzing large numbers of cells, making it a valuable tool in biomedical research and clinical studies [68,71,83].

Therefore, in this study, the cytotoxic effects of *Ilex guayusa* and *Piper marginatum* extracts on hDPSCs were evaluated using Raman spectroscopy and corroborated using fluorescence microscopy and flow cytometry to determine the safety of these extracts for oral cavity use.

## 2. Materials and Methods

An in vitro experimental study was conducted in which hDPSCs were cultured on nanobiochips placed in Corning Costar well transfer plates [84]. Subsequently, *I. guayusa* and *P. marginatum* extracts at concentrations of 10, 50, and 100 ppm were added to these plates. To assess extract cytotoxicity on the cells measurements were collected at 0, 12, and 24 h using the SERS technique [85]. The results obtained were contrasted with fluorescence microscopy and flow cytometry techniques.

### 2.1. Culture of Human Dental Pulp Stem Cells (hDPSC)

**Figure 1:**
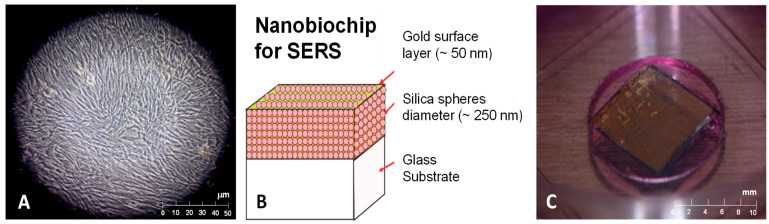
Application of nanobiochips for Raman spectroscopy on hDPSC using the SERS effect. (**A**) A 40× magnification image of the hDPSC culture, showing a monolayer formation with nearly 90% confluence. (**B**) Schematic of the nanobiochip: a colloidal crystal film, comprising 250 nm diameter silica spheres arranged on glass, and subsequently coated with a 50 nm thick gold layer. (**C**) Actual nanobiochip immersed in well transfer disks.

Human dental pulp stem cells (hDPSCs) from the Poietics™ Human Dental Pulp Stem Cells reference (LONZA) and Poietics™ DPSC BulletKit™ PT-4516 culture medium were used (Walkersville, MD, USA) [84]. A total of 5000 cells in the first thawed passage were seeded using culture medium, which included 440 mL of basal medium, 50 mL of dental pulp stem cell growth factor (DPSCGS), 10 mL of L-glutamine, 5 mL of ascorbic acid, and 0.5 mL of gentamicin/amphotericin-B (GA). Cells were distributed in two 25 cm^2^ culture flasks (250,000 cells per flask) and placed in an incubator at a temperature of 37 °C and 5% CO_2_. Media was changed the day after seeding and every two days thereafter. A cell subculture was performed when confluence reached between 80 and 90% in 48 h, and mitotic figures were observed microscopically (Figure 1A). This subculture was carried out by applying buffer, trypsinization, and neutralization.

### 2.2. Nanobiochip Elaboration

Colloidal crystals consisting of self-assembled periodic arrays of 250 nm-diameter silica spheres with a face-centered cubic structure were used. The crystals were positioned on glass substrates, and subsequently, a 50 nm gold film was deposited using high-vacuum sublimation (Figure 1B,C). The gold-coated photonic crystals provided the interface between metal and organic material with the necessary periodic roughness and nanoscopic pores to generate the surface-enhanced Raman scattering effect for signal amplification of Raman scattering by the cells under study [86,87,88,89].

### 2.3. hDPSC Seeding on Nanobiochips

**Figure 2:**
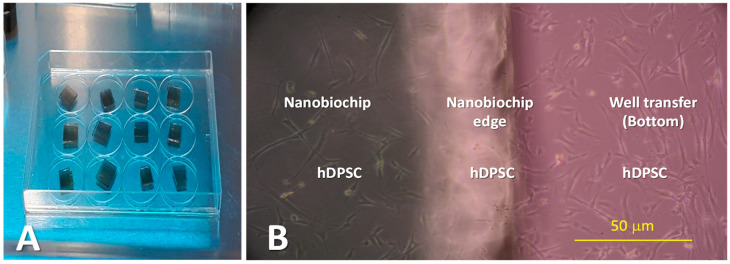
(**A**) Sterilization of nanobiochips using ethylene oxide and UV in a laminar flow hood. (**B**) Cellular adhesion (hDPSC) on the nanobiochip and bottom of the disk (40× magnification image).

The nanobiochips were previously sterilized with ethylene oxide for 24 h and ultraviolet light for 1.0 h in a laminar flow hood (Figure 2A). Once the cell confluence reached between 80 and 90%, the culture medium was removed from the 25 cm^2^ culture flasks, and to remove complex proteins and calcium and any remaining cellular debris or detritus from the culture, cells were washed with 5 mL of HEPES-buffered saline solution (HBSS). The HEPES saline solution was discarded, and the cells were detached by adding 2.0 mL of trypsin/EDTA solution (0.05% trypsin, 0.53 mM EDTA, Gibco™, Cat. No. 25300054, ThermoFischer Scientific, Grand Island, NY, USA) onto the culture plate and incubated for 5 min. Once cells were detached, as verified under the microscope, approximately 90% of the cells were observed to be round and floating on the surface. Trypsin neutralization was performed by adding 4.0 mL of trypsin-neutralizing solution (TNS). This suspension was transferred into a 15 mL Falcon tube and centrifuged at 2200 rpm for 5 min at 4 °C to obtain a cell pellet. The supernatant was discarded, and the pellet was resuspended in 1.0 mL of cell culture medium at room temperature. Subsequently, a live cell count was performed using a hemocytometer and trypan blue. For this purpose, 90 µL of trypan blue per 10 µL of suspended cells was added to an Eppendorf tube; this solution was then placed into a hemocytometer for cell counting. Once the count was completed, the number of cells to be seeded on each disk or well of the Corning Costar well transfer plate was determined. Twenty-four 35 mm-diameter well transfer disks were used, onto which the nanobiochips were deposited at the bottom, and 150,000 cells were seeded on each, with 2.0 mL of culture medium added to each disk. These cultures were incubated for 8 days with medium changes every 2 days [84]. After 8 days of culture (90% cell confluence), cellular adhesion was observed on the nanobiochips (Figure 2B), and the preparation of *I. guayusa* and *P. marginatum* extracts was then carried out.

### 2.4. Ilex guayusa and Piper marginatum Extracts

*I. guayusa* and *P. marginatum* extracts were obtained through the methodologies proposed by Sequeda-Castañeda et al. [57,90]. The plant material was acquired from Mogambo Sendero Ambiental (Municipality of Viotá, Department of Cundinamarca, Colombia) and taxonomically classified by the Herbarium of Pontificia Universidad Javeriana (HPUJ 28878-*Ilex guayusa*) and the National Herbarium of Colombia (COL575454-*Piper marginatum*). Total extracts were obtained using solid–liquid extraction using a Soxhlet apparatus and an ethanol–water solvent mixture (7:3) with a plant/solvent ratio of 1:30 for 48 h. Subsequently, to obtain a dry extract, they were concentrated using rotary evaporation at 40 °C and lyophilized. From 1.0 g of extract, stock solutions of 100 ppm of each plant species were prepared. These solutions were filtered through 0.2 μm cellulose acetate membranes. The working concentrations of 10, 50, and 100 ppm for each plant species (*I. guayusa* and *P. marginatum*) were used to determine cell viability.

### 2.5. Assessment of Plant Extract Cytotoxicity on hDPSC

To assess *I. guayusa* and *P. marginatum* extract cytotoxicity on hDPSC, cellular viability was determined using various techniques, including surface-enhanced Raman spectroscopy, fluorescence microscopy, and flow cytometry. The plant extracts were prepared at concentrations of 10, 50, and 100 ppm, which were supplemented with hDPSC media, and cellular viability was subsequently evaluated at 0, 12, and 24 h. Samples were identified as the control group (cells without extract) and the experimental group (cells with each of the extracts).

### 2.6. Cellular Viability Analysis by Raman Spectroscopy

**Figure 3:**
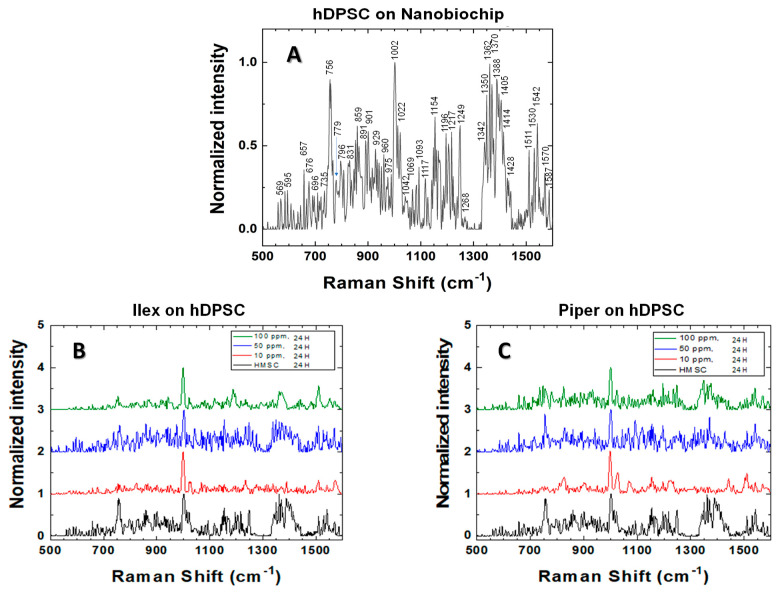
Normalized Raman spectra (SERS). (**A**) hDPSC on nanobiochips. (**B**) hDPSC + nanobiochip + *Ilex guayusa* extracts (10, 50, and 100 ppm) at 24 h. (**C**) hDPSC + nanobiochip *+ Piper marginatum* extracts (10, 50, and 100 ppm) at 24 h.

For Raman measurements, an Ocean Optics IDR-Micro 785 spectrometer equipped with a 40× optical microscope was used, allowing for the positioning of the 10-micron diameter laser beam over the cellular nucleus. The 785 nm laser beam was applied to the cells grown on the nanobiochips and immersed in their culture medium (Figure 2B). To prevent affecting their functionality, a power of 1.0 mW for 15 s was used. The characteristic Raman spectrum for these measurements was obtained in the wavenumber range of 500 to 1600 cm^−1^. Each spectrum was read 10 times consecutively, resulting in a refined average spectrum (Figure 3A–C) [70,78]. After obtaining the hDPSC cell culture, *I. guayusa* and *P. marginatum* extracts were supplemented to culture media at concentrations of 10, 50, and 100 ppm, and then Raman measurements were performed at different time intervals (0, 12, and 24 h).

### 2.7. Cellular Viability Analysis by Fluorescence Microscopy

Cellular viability was assessed using an OLIMPUS FV1000 microscope with a 10×/0.30 UPlan FLN objective and the Invitrogen™ Live/Dead kit (ThermoFisher, Eugene, OR, USA), which employs calcein and ethidium bromide to evaluate live cells and dead cells, respectively [68,69]. Once the hDPSC cell culture was obtained, *I. guayusa* and *P. marginatum* extracts were applied at 10, 50, and 100 ppm, and cell observation was conducted at different time points (0, 12, and 24 h). The culture medium was removed from each sample disk (control and experimental groups), and 150 µL of the solution composed of 10.0 mL of PBS with 5 µL of calcein (4 mM) and 20 µL of ethidium homodimer (2 mM) was added. Samples were then incubated at 25 °C for 30 min before being observed under the microscope. The percentage of live and dead cells was estimated using ImageJ 1.12 software, which allows for automated counting of all green (live) and red (dead) cells present in the samples. The parameters set in ImageJ for cell counting were as follows: (1) Open the image in ImageJ. (2) Convert to grayscale: Image > Type > 8-bit. (3) Subtract background: Process > Subtract Background. (4) Set threshold: Image > Adjust > Threshold. (5) Analyze particles: Analyze > Analyze Particles: (a) Size: 10–200 µm^2^, (b) Circularity: 0.5–1.0, (c) Show: outlines, and (d) Exclude on edges [91,92].

### 2.8. Cellular Viability Analysis Using Flow Cytometry

According to BD Biosciences instructions to evaluate cellular viability using flow cytometry, 150,000 hDPSC cells per well were cultivated in a 24-well plate [68,93]. Following eight days of cell culture, extracts (*I. guayusa* and *P. marginatum*) were added and left in contact for 0, 12, and 24 h at concentrations of 10, 50, and 100 ppm. Following the manufacturer’s instructions (BD PharmingenTM, Cat. No. 556547, BD Biosciences, San Diego, CA, USA), cells were trypsinized and stained with FITC Annexin V with Propidium Iodide (PI). The samples (0, 12, and 24 h) were then processed on a BD FACS Canto TM II flow cytometer and analyzed using FlowJoTM v10.4.2 [94].

### 2.9. Statistical Analysis

The comparison of response levels was conducted using a completely randomized design. Normal distribution was assessed using the Kolmogorov–Smirnov test. The homogeneity of variance was determined using the Levene test. To identify significant differences between group comparisons were established using ANOVA with Dunnett and Tukey post hoc tests. Transformations were performed when required (square root, square, natural logarithm, and reciprocal). A *p*-value of <0.05 was considered significant. Data without a normal distribution were evaluated using the Kruskal–Wallis and Mann–Whitney U tests [55,56]. The software used for the analyses was IBM^®^ SPSS Statistics^®^ 29.0.1, Minitab 21.1.1, and InfoStat 2020e [95,96].

## 3. Results

### 3.1. Cellular Viability Analysis Using Raman Spectroscopy

Figure 3 displays the normalized Raman spectra for the control group samples corresponding to viable hDPSC in cell culture without extracts (Figure 3A) and the experimental group samples for hDPSC with *I. guayusa extracts* (Figure 3B) and hDPSC with *P. marginatum* extracts (Figure 3C). Characteristic bands for deoxyribonucleic acid and ribonucleic acid (DNA and RNA) were observed, along with their phosphodiester bonds and the nitrogenous bases adenine (A), guanine (G), cytosine (C), thymine (T), and uracil (U) [70,97,98,99,100,101]. The DNA structure found in the cell nucleus consists of deoxyribose sugar, A, G, C, T, and phosphate groups, which are linked via phosphodiester bonds to form the chain. Similarly, the single-stranded RNA is composed of ribose sugar and the nitrogenous bases A, G, C, and U, with the latter substituting for T in DNA. Additionally, characteristic peaks for proteins, carbohydrates, and lipids were observed, compounds that constitute cell structures, including the cell membrane.

For all samples (Figure 3A–C) the **DNA** structure correlated with peaks at 729 cm^−1^ (A, ring breathing), 759 cm^−1^ (T), 765 cm^−1^ (pyrimidine ring breathing mode), 788 cm^−1^ (nucleic acid backbone), 796 cm^−1^ (phosphodiester bonds), 799 cm^−1^ (T, C), 891 and 911 cm^−1^ (deoxyribose), 1046 cm^−1^ (C-O stretching vibration), 1093 cm^−1^ (DNA β-conformation, PO_2_^−2^ phosphodiester stretching vibrations), 1157 cm^−1^ (deoxyribose, phosphate), 1183 cm^−1^ (T, C), 1256 cm^−1^ (C, A), 1294 cm^−1^ (C, T), 1470 cm^−1^ (G), 1511 cm^−1^ (A, ring breathing modes), and 1587 cm^−1^ (G, A) [74,78,98,100,101,102,103,104,105,106,107,108,109,110,111,112,113]. **RNA** correlated with peaks at 666 cm^−1^ (T-G backbone), 725 cm^−1^ (A, ring breathing mode of bases), 746 cm^−1^ (T, ring breathing mode of bases), 770 cm^−1^ (ring deformation), 782 and 785 cm^−1^ (U, T, G, ring breathing mode of bases), 802–804 cm^−1^ (ring breathing), 811 cm^−1^ (O-P-O stretching), 813 cm^−1^ (ribose), 828 cm^−1^ (O-P-O phosphodiester stretching), 867 cm^−1^ (ribose), 915 cm^−1^ (ribose), 974 cm^−1^ (ribose), 1010 cm^−1^ (C-H wagging), 1054 cm^−1^ (ribose C-O vibration), 1120 cm^−1^ (C-O band of ribose), 1208 cm^−1^ (ring breathing modes of the bases), 1220 cm^−1^ (T, A), 1240 cm^−1^ (ribose), 1257 cm^−1^ (A, T), 1263 cm^−1^ (T, A, ring breathing modes of the bases), 1318 cm^−1^ (G, ring breathing modes of the bases), 1342 cm^−1^ (G), 1373 cm^−1^ (T, A, G, ring breathing modes of the bases), 1420 cm^−1^ (G, A), 1570 cm^−1^ (G, A, ring breathing modes of the bases), and 1587 cm^−1^ (G, A) [74,78,98,100,101,102,103,104,105,106,107,108,109,110,111,112,113].

For **proteins**, bands were observed at 621 cm^−1^ (C-C twist Phe), 645 cm^−1^ (C-C twist Tyr), 756 and 760 cm^−1^ (Trp, ring breath), 828 cm^−1^ (Tyr), 834 cm^−1^ (Tyr, ring breathing vibration), 854 cm^−1^ (Tyr), 858 cm^−1^ (Tyr, fermi resonance double with 834 cm^−1^), 958 cm^−1^ (Phe, C-C stretching), 1002 cm^−1^ (Phe), 1117 cm^−1^ (benzoid ring deformation), 1154 cm^−1^ (C-C, C-N protein stretching), 1167 cm^−1^ (iLeu), 1245 cm^−1^ (amide III, β-sheet), and 1402 cm^−1^ (Arg, Cβ rocking) [74,78,98,100,101,102,103,104,105,106,107,108,109,110,111,112,113]. Characteristic peaks for **lipids** were identified at 717 cm^−1^ (CN^+^(CH_3_)_3_, stretching), 877 cm^−1^ (C-C-N^+^, symmetric stretching), 980 cm^−1^ (C-H bend), 1032 cm^−1^ (CH_2_-CH_3_ bending modes), 1069 cm^−1^ (chain C–C stretching), 1078 cm^−1^ (C-C, C-O vibration), 1123 cm^−1^ (C-C stretching), 1168 cm^−1^ (C=C, C-OH vibration), 1220 cm^−1^ (C-H bend), 1249 cm^−1^ (C-H deformation), 1268 cm^−1^ (CH_2_ in-plane deformation), 1270 cm^−1^ (C=C groups in unsaturated fatty acids), 1301 cm^−1^ (C-H deformation vibrations), 1388 cm^−1^ (CH_3_ vibration), 1393 cm^−1^ (CH rocking), 1398 cm^−1^ (CH_2_ deformation), 1405 cm^−1^ (CH deformation), 1420 cm^−1^ (CH_2_ scissoring), 1428 cm^−1^ (CH_2_ bending), 1449 cm^−1^ (C-H deformation vibrations), 1525 cm^−1^ (in-plane vibrations of the conjugated -C=C-), and 1660 cm^−1^ (C=C stretching) [74,78,98,100,101,102,103,104,105,106,107,108,109,110,111,112,113]. Peaks for **carbohydrates** were recognized at 877 cm^−1^ (C-O-H ring vibration), 937 cm^−1^ (C-O-H glycoside vibration), 1060 cm^−1^ (C-O, C-C, stretching), 1128 cm^−1^ (C-O stretching), 1342 cm^−1^ (CH deformation), and 1428 cm^−1^ (CH deformation) [74,78,97,98,100,101,102,103,104,105,106,107,108,109,110,111,112,113]. For **phospholipids**, the band was identified at 719 cm^−1^ (C-C-N^+^ symmetric stretching). **Phosphatidylcholine,** the major constituent in cellular membranes was observed at 1032 cm^−1^ (CH_2_-CH_3_ bending modes of phospholipids), 1078 cm^−1^ (C-C or C-O stretching mode), and 1268 cm^−1^ (C-H vibration) [74,78,98,100,101,102,103,104,105,106,107,108,109,110,111,112,113].

### 3.2. Cellular Viability Analysis Using Fluorescence Microscopy

**Figure 4:**
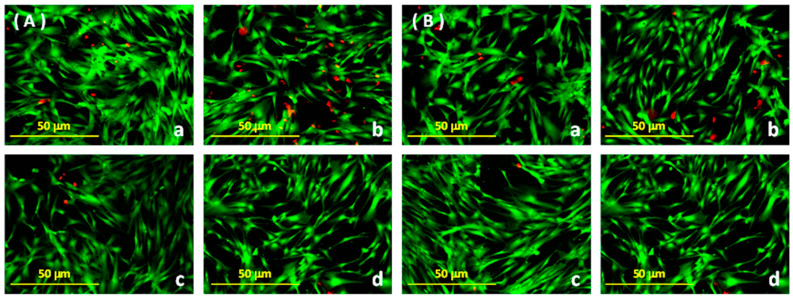
Fluorescence microscopy using a 10×/0.30 objective lens. Extracts of *Ilex guayusa* (**A**) and *Piper marginatum* (**B**) on Poietics™ Human Dental Pulp Stem Cells (hDPSC) on the 8th day of culture. The green cells represent the living cells (stained with calcein), while the red cells represent the dead cells (stained with ethidium bromide). hDPSC control without extract at 24 h (**a**). hDPSC with the extract at 10 ppm at 24 h (**b**). hDPSC with the extract at 50 ppm at 24 h (**c**). hDPSC with the extract at 100 ppm at 24 h (**d**).

Images corresponding to hDPSC cellular viability when in contact with *I. guayusa* and *P. marginatum* extracts at different concentrations over several hours (between 0 and 24 h of exposure) are presented in Figure 4.

Table 1 displays hDPSC viability percentages obtained using fluorescence microscopy for *I. guayusa* and *P. marginatum* extracts at different concentrations during a time interval from 0 to 24 h of exposure.

The data from Table 1 were analyzed using factorial ANOVA. It was conducted considering the factors and their corresponding levels as plant (*I. guayusa* and *P. marginatum*), concentration (0, 10, 50, and 100 ppm), and time (0, 12, and 24 h), as well as the interactions between factors (plant–concentration, plant–time, and concentration–time). For each plant, 0 ppm at 0 h extract was used as the control. The plant type factor resulted in significant differences (*p* = 0.016), indicating that significant differences in hDPSC cellular viability were observed when using *I. guayusa* and *P. marginatum* extracts. These differences are due to the fact that they correspond to plants of different families, genera, and species. Other factors, such as concentration (*p* = 0.171) and time (*p* = 0.256), did not have a significant effect on hDPSC cellular viability. Similarly, plant–concentration interactions (*p* = 0.971), plant–time (*p* = 0.565), and concentration–time (*p* = 0.675) did not result in significant differences. In summary, cellular viability when comparing *I. guayusa* and *P. marginatum* extract effect showed significant differences. However, they did not induce cytotoxicity in the hDPSC cell line within the evaluated time and concentration ranges.

### 3.3. Cellular Viability Analysis Using Flow Cytometry

Table 2 provides hDPSC’s viability percentages obtained through flow cytometry for *I. guayusa* and *P. marginatum* extracts at different concentrations during 24 h of exposure.

The data from Table 2 were analyzed using factorial ANOVA. Factors and their corresponding levels included plant (*I. guayusa* and *P. marginatum*), concentration (0, 10, 50, and 100 ppm), and time (0, 12, and 24 h), along with interactions between factors (plant–concentration, plant–time, and concentration–time). Concentrations of 0 and 50 ppm at 0 h for each plant extract were considered controls, respectively. No significant differences were found for the factors plant (*p* = 0.457), concentration (*p* = 0.919), and time (*p* = 0.544), as well as for the interactions plant–concentration (*p* = 0.923), plant–time (*p* = 0.892), and concentration–time (*p* = 0.981). Therefore, both *I. guayusa* and *P. marginatum* extracts did not induce a cytotoxicity effect on the hDPSC cell line at concentrations of 0, 10, 50, or 100 ppm.

## 4. Discussion

Raman spectroscopy is a non-invasive and non-destructive technique that does not require prior sample preparation. This has led to its increased utilization in in vitro, ex vivo, and in vivo analyses of cells and tissues, providing a high degree of molecular specificity and generating a unique fingerprint for each type of sample [74,78,100,114,115,116,117,118]. Recent studies employing Raman spectroscopy to monitor DNA, protein, and lipid damage induced by toxic agents have demonstrated that this technique is rapid, cost-effective, and applicable in clinical settings for monitoring the cellular effects of toxic agents [119]. The Raman technique allows for cellular component visualization based on their biochemical composition, including nucleic acids, proteins, mitochondria, lipids, and carbohydrates [78,120]. Despite its ultra-sensitivity, at low concentrations, Raman spectroscopy is susceptible to dispersion per molecule, causing interference in the detection of analytes. In biological samples, in which compounds are often at physiological trace levels, Raman signals may overlap with background fluorescence, complicating analyte identification. Furthermore, biological samples must be excited for short durations using wavelengths above 400 nm and with a power density below 0.5 mW/μm^2^ to prevent cellular mutation or apoptosis [117,118,121]. To address these limitations, surface-enhanced Raman spectroscopy has been developed, improving the Raman signal when molecules contact a nanostructured metallic surface, typically gold, silver, or copper [122]. This enhancement results from the electronic cloud interaction of the metallic nanoparticles with surface electrons of sample molecules under the incident laser light electric field. This interaction is augmented by charge transfer effects and “hot spots” (regions between nanoparticles), increasing the Raman signal by 10 to 15 orders of magnitude [72,122,123,124].

In this study, a material (nanobiochip) was developed using silica nanoparticles (250 nm diameter) coated with a periodically roughened 50 nm-thick gold film (Figure 1C). The SERS effect was observed with this nanobiochip, when the 785 nm laser penetrated the hDPSC (Figure 2B) and the laser’s electric field excitation coupled with the metal plasmons, generating a more intense local electric field than that of the laser itself. This led to the enhanced polarization of the molecules within the hDPSC, resulting in signal augmentation, as recorded in the Raman spectra (Figure 3A–C).

From the hDPSC Raman spectrum (Figure 3A), peaks at 729 cm^−1^ (A) and 765 cm^−1^ (C and T) served as references for DNA, while peaks at 770 and 802 cm^−1^ (U) were used for RNA. The peak at 1002 cm^−1^, corresponding to the phenylalanine ring, indicates cellular death. Peaks at 1096 cm^−1^ (phosphodiester bonds) are sensitive to alterations in the DNA structure [78,99,100,102,103,125,126]. Peaks at 1257 cm^−1^ and 1656 cm^−1^ indicate structural changes in proteins. The decrease in these peak intensities is associated with the destruction or degradation of DNA, RNA, and proteins [99,100,103].

hDPSC Raman spectra exposed to *I. guayusa* and *P. marginatum* extract comparison revealed no significant changes (*p* > 0.05) in the Raman signal between plants, time, and evaluated concentrations, indicating that the cells were viable without physiological alterations or cell death from irreversible morphological, functional, and chemical changes; thus, no cytotoxicity from the extracts on hDPSC was observed. Notably, in samples with *I. guayusa* (10, 50, and 100 ppm), a greater decrease at 12 h, in comparison with 24 h, was observed, as evidenced by the Raman intensity peaks at 765 cm^−1^ (C and T) and 1096 cm^−1^ (marker for DNA phosphodiester bond stability). However, when these samples were compared to the 0 ppm control (12 and 24 h), the decrease in Raman intensity was not significant (*p* > 0.05), suggesting that the decrease in the DNA damage marker peak was not important, regardless of whether hDPSC were in media with extracts for longer periods. This effect can be attributed to DNA repair mechanisms that reverse damage by increasing transmembrane pump activity to remove extracts from the cell and confer hDPSC a degree of resistance to extracts, as described by McKeegan et al. 2003 and others [127,128].

Moreover, autophagy might explain, to a lesser extent, the cellular death mechanism (apoptosis) activated in some cells as part of their initial adaptation to stress [98]. Once this adaptation occurs, the activated autophagy is interrupted, and cells can return to their original state. This coincides with the observation that at both 12 and 24 h of extract exposure, most cells retained their morphology and remained adherent, and only a few were floating, demonstrating signs of possible apoptosis [129]. This low percentage of cellular death for the concentrations used was also detected using the fluorescence microscopy technique (Table 1, Figure 4) and with flow cytometry (Table 2), indicating that the extracts did not induce cytotoxicity in hDPSC.

Using fluorescence microscopy, it was observed that hDPSC cell viability decreased when in contact with *I. guayusa* extract at 10 ppm for 12 h, but viability increased at 24 h (Table 1). As mentioned earlier, this effect may be due to the DNA repair mechanism through increased activity of transmembrane pumps or due to the autophagy effect [98,127,128,129]. Ultimately, this effect did not affect cell viability, even at the highest concentration and longest extract exposure time, demonstrating that extracts did not exert a cytotoxic effect on hDPSC.

The flow cytometry results (Table 2) also demonstrated no cytotoxic effect on hDPSC with plant extracts at the concentrations and contact times evaluated. Collectively, extracts did not have a cytotoxic effect on hDPSC.

Regarding hDPSC cell viability, when contrasting Raman spectroscopy results with those of fluorescence microscopy and flow cytometry, no differences were observed for extract concentrations from each plant within the evaluated time interval. Furthermore, for studying live cells in vitro, the Raman spectroscopy technique’s advantages, compared to fluorescence microscopy and flow cytometry, are as follows: cell fixation or staining is not required, it does not destroy the cells, and it is capable of real-time cell monitoring [115,125]. Consequently, cell viability results obtained using the three techniques indicate that *I. guayusa* and *P. marginatum* extracts at the evaluated concentrations and time were not toxic.

Based on the aforementioned results in this study and by comparing the advantages and limitations of Raman spectroscopy, fluorescence microscopy, and flow cytometry in determining cell viability, we conclude the following for Raman spectroscopy: (1) it does not require staining or fluorescent markers, allowing for non-invasive cell analysis; (2) it provides detailed chemical information on the cell’s molecular composition, helping characterize their metabolic and functional state; (3) it is compatible with live or fixed biological samples, enabling the analysis of both live and dead cells; (4) it requires specialized equipment and technical expertise for operation and data interpretation; (5) it has lower sensitivity compared to techniques like fluorescence microscopy for detecting specific cellular events; and (6) it may be slower in data acquisition compared to other techniques. For fluorescence microscopy we determined that (1) it is a highly sensitive and specific technique for detecting fluorescent markers, allowing for specific cellular event study; (2) it enables the direct visualization of cellular processes in real-time with high spatial resolution; (3) it is widely used in biological and medical research, with a variety of techniques and applications available; (4) it requires the use of specific fluorescent markers, which can affect cell viability and interpretation of results; (5) it may be limited in the penetration of thick samples or dense tissues; and (6) it is not suitable for the analysis of opaque or autofluorescent samples. For flow cytometry (fluorescent activated cell sorter): (1) it allows for the rapid and quantitative analysis of large populations of individual cells; (2) it offers versatility in detecting multiple cellular parameters, such as size, complexity, viability, and protein expression; (3) it is compatible with a variety of fluorescent markers and cell viability assays; (4) it requires specific sample preparation, including the use of fluorescent markers and cell fixation; (5) it may not provide the spatial resolution of fluorescence microscopy; and (6) it may be less suitable for complex sample analysis of solid tissues due to the need to disaggregate cells into a suspension. In summary, each technique has its own advantages and limitations in determining cell viability. The choice of the most suitable technique will depend on the specific needs of the experiment, sample characteristics, and desired cellular information.

We employed hDPSC because these cells have demonstrated phenotypic and functional characteristics very similar to those of mesenchymal stem cells derived from bone marrow and are considered one of the key cells in bone and tooth regeneration engineering [112,130,131]. It has been reported that human dental pulp stem cells (DPSC) differentiate into many different lineages, including chondrogenic, osteogenic, adipogenic, and neural lineages [110,132,133]. hDPSCs are isolated from a donor’s adult third molars collected during the extraction of wisdom teeth [102]. Furthermore, considering that dentinal tubules extend from the pulp to the dental enamel [134,135,136] and participate in fluid diffusion toward the pulp [134,137,138], it was considered necessary to analyze the effects of *I. guayusa* and *P. marginatum* extracts on dental pulp. Moreover, in enamel alterations such as AI, in which fluid diffusion presents an additional increase in dentin permeability due to a decrease in its quality or the total loss of enamel, dentin becomes more conductive and exposes the dentin–pulp complex more easily [136]. Additionally, odontoblasts are the first pulp cells to respond to exogenous stimuli [134,139,140], but due to their post-mitotic phenotype, they are difficult to culture [141,142]. Therefore, toxicological studies on these cells have been limited, and cells such as primary gingival fibroblasts, primary mouse cells, immortalized cell lines, and undifferentiated human mesenchymal stem cells have been used [143,144,145,146]. The latter have characteristics of adherence in cultures [131], ease of extraction, low mortality, and a high capacity for differentiation and proliferation [130,132].

Previous studies conducted by the Phytochemistry Universidad Javeriana Research Group (GIFUJ) systematized information on *I. guayusa* and *P. marginatum* in the form of monographs [49,52], in which taxonomy data, ethnobotany, geographical distribution and habitat, ecology, phytochemistry, phytosanitary aspects, and biological activity were compiled. Similarly, GIFUJ and the Center for Dental Research (CIO) have evaluated other types of biological activities, such as anticariogenic, antiperiodontal, and a potential remineralization effect on dental enamel [56,57,90,147]. Plant extract compounds related to these biological activities correspond to secondary metabolites, such as phenols, polyphenols, flavonoids, proanthocyanidins, and tannins, as well as mineral compounds like phosphates and orthophosphates [48,57,90].

To our knowledge, this is the first study evaluating *I. guayusa* and *P. marginatum* extracts cytotoxicity on hDPSC. In addition, interdisciplinary work between research groups from the Departments of Physics, Chemistry, and Dentistry of the Pontificia Universidad Javeriana shows the potential development of a nanobiochip to qualitatively evaluate cytotoxicity using Raman spectroscopy as an alternative technique to others (FM and FACS). Additionally, a non-invasive hDPSC analysis was performed without the need for staining or using fluorescent markers, providing a detailed molecular composition to characterize their metabolic and functional state in living or dead cells. Further studies need to be performed on the development and implementation of the nanobiochip to generate quantitative data. In addition, to validate *I. guayusa* and *P. marginatum* extract use in toxicity studies, animal models such as mice and rats are also required in the following phases (preclinical and clinical) to develop a product for oral cavity use.

## 5. Conclusions

*Ilex guayusa* and *Piper marginatum* extract cytotoxicity effects on human dental pulp stem cells (hDPSC) were evaluated using surface-enhanced Raman spectroscopy (SERS), fluorescence microscopy, and flow cytometry. The results showed minimal and non-dose-dependent cytotoxic effects, suggesting their biocompatibility. These findings indicate that these extracts hold promise for future research in regenerative medicine and tissue engineering applications. Further studies are needed to identify the bioactive compounds and their therapeutic applications in dental and biomedical research.

## Figures and Tables

**Table 1:** Cellular viability (%) using fluorescence microscopy *.

**Hours**	***Ilex guayusa***			
	0 ppm *	**10 ppm**	**50 ppm**	**100 ppm**
0	83.3 ± 27.6	87.8 ± 27.4	78.8 ± 31.1	72.7 ± 33.5
12	85.2 ± 21.9	69.2 ± 28.0	91.9 ± 24.2	92.7 ± 23.1
24	89.1 ± 27.5	91.5 ± 24.0	95.2 ± 22.1	93.8 ± 22.2
**Hours**	***Piper marginatum***			
	0 ppm *	**10 ppm**	**50 ppm**	**100 ppm**
0	94.7 ± 27.7	95.8 ± 25.6	93.4 ± 23.2	92.4 ± 26.5
12	96.3 ± 25.6	90.6 ± 20.2	94.1 ± 21.3	95.2 ± 22.3
24	97.1 ± 21.5	95.2 ± 21.6	97.5 ± 18.7	96.1 ± 16.7

* Average ± standard deviation $\left(\bar{x}\pm s\right)$, n = 9. Control: hDPSC without extract.

**Table 2:** Cellular viability (%) using flow cytometry *.

**Hours**	Controls *			
	**0 ppm**		**50 ppm**	
0	90.4 ± 19.9		89.6 ± 21.8	
12	88.1 ± 18.5		89.4 ± 19.0	
24	89.8 ± 15.9		88.9 ± 22.1	
**Hours**	***Ilex guayusa***			
	0 ppm *	**10 ppm**	**50 ppm**	**100 ppm**
0	88.4 ± 21.2	87.3 ± 16.5	90.1 ± 23.2	89.7 ± 22.2
12	87.6 ± 23.5	83.8 ± 19.6	88.9 ± 18.4	79.9 ± 17.1
24	90.1 ± 17.4	85.6 ± 17.6	91.8 ± 20.0	91.5 ± 19.8
**Hours**	***Piper marginatum***			
	0 ppm *	**10 ppm**	**50 ppm**	**100 ppm**
0	88.4 ± 21.2	88.9 ± 19.0	90.4 ± 15.4	89.6 ± 15.1
12	87.6 ± 23.5	85.6 ± 19.9	91.8 ± 14.5	91.5 ± 11.4
24	90.1 ± 17.4	92.5 ± 19.3	94.4 ± 20.1	91.7 ± 19.4

* Average ± standard deviation $\left(\bar{x}\pm s\right)$, n = 9. Control: hDPSC without extract.

## Data Availability

All data and materials used in this research are available from the corresponding author upon reasonable request.
